# BTS1-knockout *Saccharomyces cerevisiae* with broad-spectrum antimicrobial activity through lactic acid accumulation

**DOI:** 10.3389/fcimb.2025.1494149

**Published:** 2025-01-31

**Authors:** Liu Cong, Yuan Zhou, Yu Zhang, Shanshan Mao, Chaoqun Chen, Liying Wang, Xiao Li, Zuo Zhang, Zuobin Zhu, Ying Li

**Affiliations:** ^1^ School of Medical Technology, Xuzhou Medical University, Xuzhou, Jiangsu, China; ^2^ Department of Clinical Laboratory, Xuzhou Central Hospital, Xuzhou, Jiangsu, China; ^3^ Department of Genetics, Xuzhou Medical University, Xuzhou, Jiangsu, China

**Keywords:** probiotics, *Saccharomyces cerevisiae*, multidrug resistant pathogens, broadspectrum antibacterial activity, antimicrobial metabolites

## Abstract

Bacterial infections pose significant threats to human health, and prudent antibiotic use remains a key strategy for disease treatment and control. However, a global escalation of drug resistance among pathogenic bacteria presents a formidable challenge. Probiotics have emerged as a promising approach to combating pathogenic bacterial infections. In this study, we investigated the antibacterial activity of BTS1-knockout (BTS1-KO) *Saccharomyces cerevisiae*. Our findings demonstrate its effective inhibition of pathogen growth as evidenced by Minimum inhibitory concentration (MIC) assays, growth curves, bacteriostatic spectrum analyses and co-culture experiments. Additionally, it significantly impedes *Escherichia coli* and *Staphylococcus aureus* biofilm formation. Moreover, BTS1-KO *S. cerevisiae* exhibits low haemolytic activity, acid resistance, resistance to high bile salt concentrations, high auto-aggregation capacity and high co-aggregation capacities with pathogenic bacteria. Moreover, infected larvae treated with BTS1-KO *S. cerevisiae* in *Galleria mellonella*-*E. coli* (*in vivo*) and *G. mellonella*-*S. aureus* (*in vivo*) infection models showed significantly prolonged survival times. Mechanistic investigations revealed that BTS1-KO *S. cerevisiae* primarily produced lactic acid via metabolism, thereby lowering the environmental pH and inhibiting pathogenic bacterial growth. In summary, our study underscores the probiotic potential of BTS1-KO *S. cerevisiae*, offering broad-spectrum antibacterial activity *in vitro* and *in vivo* with low toxicity. This highlights BTS1-KO *S. cerevisiae* as a promising probiotic candidate for clinical prevention and control of bacterial infection.

## 1 Introduction

Bacterial infections represent a significant global health threat, as highlighted in the World Health Organisation’s ‘Top Ten Global Health Threats’ report, due to their elevated morbidity and mortality rates (EclinicalMedicine, 2021). The misuse of antibiotics has driven a rise in antibiotic-resistant pathogenic microbes, resulting in approximately 1 million deaths annually from untreatable bacterial infections, posing a grave threat to public health and safety (Subramaniam and Girish, 2020). For example, polymyxin is regarded as the last line of defence against multidrug-resistant gram-negative infections, and *Escherichia coli* carrying the polymyxin resistance gene *mcr-1* (*E. coli* (*mcr-1*)) can transmit between animals and humans, increasing the difficulty of treating the infection (Zhang et al., 2021). Methicillin-resistant *Staphylococcus aureus* (MRSA) is a prevalent and virulent pathogenin clinical settings due to its broad-spectrum resistance to antibiotics such as *β*-lactams, cephalosporins, aminoglycosides, macrolides, tetracyclines, fluoroquinolones, sulfonamides and rifampicin (Oteo and Belén Aracil, 2015; Jain et al., 2023). By 2050, antibiotic resistance is projected to cause 10 million deaths annually (Palavecino, 2020). Urgent measures are needed to develop novel therapeutic agents or strategies to address this clinical challenge.

Probiotics, defined as living microorganisms conferring beneficial effects upon the host when administered in adequate amounts, offer a promising alternative to antibiotics (Kim et al., 2019; Oniszczuk et al., 2021). Recently, the use of probiotics to prevent or control bacterial infections has garnered attention. Numerous studies reports that probiotics can inhibit the proliferation and virulence characteristics of various pathogenic bacteria. For example, yeast *Saccharomyces boulardii* can alleviate intestinal infections caused by *E. coli*, *S. aureus* and *Salmonella* (Pais et al., 2020); *Lactobacillus curvatus* protects the host from urinary tract pathogenic *E. coli* infection by promoting type I interferon production in the bladder epithelial cells, enhancing phagolysosome maturation and histone D production, along with enhanced acidification (Song et al., 2022). Additionally, *Lactococcus lactis* detects CAI-1, a population-sensing signal of *Vibrio cholerae*, thereby inhibiting its colonisation in the intestine (Mao et al., 2018).

*Saccharomyces cerevisiae*, widely employed in oral vaccine development and medical engineering vectors, exhibits antimicrobial properties with *S. boulardii*, indicating potential as a prebiotic agent (Kaźmierczak-Siedlecka et al., 2020). In this study, we utilised the yeast *S. cerevisiae* gene-knockout collection (YKOC) to screen strains with antibacterial activity. BTS1-knockout (BTS1-KO) *S. cerevisiae*, demonstrating effective antibacterial activity, was selected for further characterisation of its antibacterial activity, toxicity profile and probiotic characteristics both *in vitro* and *in vivo*. Our findings elucidate the preliminary mechanism of BTS1-KO *S. cerevisiae* against pathogenic bacteria and provide a theoretical basis for its potential clinical application as a probiotic agent.

## 2 Materials and methods

### 2.1 Strains

*S. cerevisiae* single-gene deletion strain and BY4743 parent strain were obtained from YKOC, which was purchased from Invitrogen in 2014. All strains were stored at −80°C in a preservation solution containing 20% glycerol (v/v) (Sigma-Aldrich). Before the experiment, the strains were inoculated twice on Yeast Extract Peptone Dextrose (YPD)ager plates (yeast extract 1%, peptone 2%, glucose 2%, and agar 2%) (Qingdao Hope Bio-technology Company) and incubated at 30°C. Single colonies were inoculated into the YPD broth (2% tryptone, 1% yeast extract, and 2% glucose) and cultured overnight at 30°C, 200 r/min. *S. boulardii* CNCM I-745 (imported drug registration No. s5288500) was ordered in French Encyclopedia Pharmaceutical Factory; *E. coli* ATCC25922, *E. coli* (*mcr-1*) 12-2, *S. aureus* ATCC29213, *S. aureus* ATCC25923, MRSA 1668, *Pseudomonas aeruginosa* ATCC27853, *P. aeruginosa* 1554, *Klebsiella pneumoniae* ATCC700603, *K. pneumoniae* ATCC1706, *K. pneumoniae* ATCC1705, *K. pneumoniae* 2118, *Acinetobacter baumannii* 21-1 and *Salmonella* Typhimurium SL1344 were donated by the Laboratory of Affiliated Hospital of Xuzhou Medical University. *Lactobacillus johnsonii* D-SM 10553 freeze-dried powder (SHBCC, China) was solubilised with 0.5 mL of de Man Rogosa and Sharpe (Falsen et al., 1999) (MRS) medium (Qingdao Hope Bio-technology Company), then the bacterial solution was applied to MRS solid plates, and single bacterial colonies appeared after about 24 h. Subsequently frozen stocks of *L. johnsonii* (in MRS medium with 20% glycerol) were prepared, stored at -80°C for further experiments. *E. coli*, *S. aureus*, *P. aeruginosa*, *K. pneumoniae*, *A. baumannii* and *S.* Typhimurium were incubated in Luria-Bertani (LB)broth (Tryptone 10.0 g, Yeast Extract 5.0 g, NaCl 10.0 g per litre) (Shanghai Sangon Biotech Company) at 37°C overnight. *L. johnsonii* was incubated in MRS broth overnight at 37°C under anaerobic conditions.

### 2.2 Preparation of cell-free supernatant from *S. cerevisiae*

The *S. cerevisiae* single-gene deleted strain and the BY4743 parent strain were inoculated into YPD broth and cultured at 30°C for 16 h in a shaker at 200 rpm. After incubation, the samples were centrifuged at 4000×g at 4°C for 10 min. Then, the supernatants were filtered by a syringe filter (0.22-μm pore size), and then stored at -80°C for later use, in which CFS of BY4743 was used as a control.

### 2.3 Screening experiments for CFSs with bacteriostatic properties

CFS with antibacterial activity was screened by 96-well plate method. A volume of 90 μL of CFS of deletion strain was mixed with 10 μL of bacterial liquid (1×10^8^ CFU/mL) in each well of 96-well plate, and CFS of BY4743 was used as control group. The pathogenic bacteria used in this experiment are *E. coli* ATCC25922, *E. coli* (*mcr-1*) 12-2, *S. aureus* ATCC29213 and MRSA 1668. The well plates were cultured at 37°C for 18-24 h, and the absorbance at 600 nm was measured by enzyme-labelled instrument. The ratio of OD value of each well divided by OD value of the control group was regarded as its growth rate, and the strains with inhibition rate (1- growth rate) greater than 80% were regarded as having antibacterial activity.

### 2.4 Minimum inhibitory concentration of CFS

The MIC method was employed to assess the antibacterial activity of CFS of BTS1-KO *S. cerevisiae*. Pathogenic bacteria cultured overnight were diluted in the Mueller Hinton (MH) broth medium (Qingdao Hope Bio-technology Company). Various concentrations of CFS and bacterial suspensions were added to 96-well plates, resulting in a final volume of 100 μL per well and a bacterial concentration of 1×10^6^ CFU/mL, compared to wells without CFS. The pathogenic bacteria included *E. coli* ATCC25922, *E. coli* (*mcr-1*) 12-2, *S. aureus* ATCC29213 and MRSA 1668. Then the plates were cultured at 35°C for 24 h, and the lowest CFS concentration to inhibit bacterial growth or inhibit 80% of bacterial cells as MIC.

### 2.5 Antibacterial activity of CFS against different bacteria

The antimicrobial activity of CFS against various bacterial strains was determined according to the screening method, and the inhibitory activity of CFS of *S. boulardii* and *L. johnsonii s* was used as a control.

### 2.6 Growth curve determination

Overnight cultures of pathogenic bacteria were diluted in LB broth. Experimental groups were prepared by mixing bacterial suspensions with 90% CFS, 50% CFS or 25% CFS, while the control group was mixed with YPD broth, and a blank control group was mixed with sterile water. The pathogenic bacteria were *E. coli* ATCC25922, *E. coli* (*mcr-1*) 12-2, *S. aureus* ATCC29213 and MRSA 1668. Initial optical density at 600 nm (OD_600_) was measured using an enzyme-labelled instrument (Thermo Fisher Scientific) and then cultured at 37°C and 200 rpm. Samples of 100 μL were taken every 2 h to determine OD_600_ and construct growth curves.

### 2.7 Biofilm inhibition assay

The inhibition of biofilm formation by CFS was evaluated using the crystal violet (CV) method (Xu et al., 2016). Then, 90%, 50% or 25% of CFS concentrations were mixed with bacterial suspensions in 96-well plates (experimental group), while control groups were mixed with YPD broth and incubated at 37°C for 24 h. The pathogenic bacteria were *E. coli* ATCC25922, *E. coli* (*mcr-1*) 12-2, *S. aureus* ATCC29213 and MRSA 1668. After incubation, the cell suspension was washed twice with 150 μL sterile water. Biofilm cells were dried at 37°C for 30 min and then stained with 0.1% 150 μL CV solution for 30 min. Subsequently, the excess CV solution was gently washed away with sterile distilled water. and 150 μL of solution (30% methanol and 10% acetic acid) was added to each well to dissolve the CV solution. Finally, the OD_570_ was measured using an enzyme-labelled instrument. The following equation was used to calculate the biofilm inhibition rate (%):


$$\text{Biofilm inhibition rate}\left(\%\right)=\left(1-{\text{OD}}_{\text{treatment}}/{\text{OD}}_{\text{control}}\right)\times 100$$

### 2.8 Scanning electron microscopy analysis

Suspensions of pathogenic bacteria were co-cultured with varying amounts of CFS in 24-well plates, with sterile polystyrene discs added to wells. Control wells contained bacteria without CFS. After 24 h of incubation at 37°C, planktonic bacteria were removed, washed thrice with phosphate buffer solution (PBS) (Shanghai Sangon Biotech Company), fixed with 1% osmium acid solution at 4°C for 2 h and dehydrated with an ethanol gradient (50%, 70%, 80%, 90% and 95%) for 10-20 min. Then, add tert-butyl alcohol to infiltrate for 2 h. Finally, the film was fixed on the surface of the wafer after carbon dioxide critical drying and ion sputtering, revealing a golden yellow surface, and the film was observed under an SEM (American FEI Company).

### 2.9 Determination of adhesion ability

Overnight cultures of *E. coli* and *S. aureus* were diluted in LB broth. Bacterial suspensions were mixed with 50% CFS and incubated in glass tubes at 37°C for 24 h. The control group was mixed with the same amount of YPD broth. The glass tube was placed at an angle of 30 and incubated at 37°C for 24 h. After incubation, the floating cells were retained, and the adhered cells were collected, washed with PBS, resuspended in PBS buffer and their OD_600_ was measured (OD _adhesion)_. Then, the planktonic cells were mixed with the adherent cells, washed with PBS and resuspended and the OD_600_ of the mixed cells was measured (OD _mixture_). Adhesion capacity (%) was calculated using the following formula:


$$\text{Adhesion ability}\left(\%\right)=\left({\text{OD}}_{\text{adhesion}}/{\text{OD}}_{\text{mixture}}\right)\times 100$$

### 2.10 Extracellular polysaccharides production determination

Overnight cultures of *E. coli* and *S. aureus* were prepared in LB broth. The bacterial suspension was mixed with 50% CFS, while the control group received an equivalent volume of YPD broth and was cultured at 37°C for 24 h. Following incubation, it was centrifuged at 8000 ×g for 10 min at 4°C, washed twice with PBS, resuspended with 0.9% saline (1 mL) and then mixed with 5% phenol and 5% sulfuric acid in equal volumes and incubated in darkness for 1 h. The OD_490_ was measured to quantify EPS production using the following equation:


$$\text{EPS quantification}\left(\%\right)=\left({\text{OD}}_{\text{treatment}}/{\text{OD}}_{\text{control}}\right)\times 100$$

### 2.11 Hydrophobicity assay

Overnight cultures of *E. coli* and *S. aureus* were prepared in LB broth. The experimental group received 50% CFS mixed with bacterial suspension, while the control group received an equal volume of YPD broth and was cultured at 37°C for 24 h. After incubation, the cell suspension was centrifuged at 17,709× g at 4°C for 5 min, washed twice with PBS and resuspended in fresh PBS. The OD_600_ was adjusted to 0.5 ± 0.05 (OD _initial_). Then, 1.2 mL of the cell suspension was transferred to a glass test tube, mixed with n-octane (0.3 mL) and vortexed for 3 minutes before standing for 15 minutes. The lower water phase was collected, and the OD_600_ (OD _treatment_) was measured.

### 2.12 Establishment of bacteria-*S. cerevisiae* culture model

To assess the inhibitory effort of live BTS1-KO *S. cerevisiae* on *E. coli* and *S. aureus* cells, bacteria-*S. cerevisiae* co-culture model was established. Overnight cultures of *S. cerevisiae* and pathogenic bacteria were washed twice with PBS and diluted to 10^7^ CFU/mL in YPD and LB broth medium, respectively. Co-culture groups were prepared by mixing *S. cerevisiae* and pathogenic bacteria at a 1: 1 ratio (1 mL each). The pathogen culture was mixed with YPD broth (1 mL each) as the single culture group, while the blank group consisted of pathogenic bacteria mixed with sterile water (1 mL each). Cultures were incubated at 37°C and 200 rpm. The cells in the co-culture group and pathogen single culture group were counted on an LB agar plate supplemented with 10 mg/mL amphotericin B (Sigma-Aldrich). The experiment was performed in triplicate.

### 2.13 Probiotic potential of BTS1-KO *S. cerevisiae*

#### 2.13.1 Determination of haemolytic activity

BTS1-KO *S. cerevisiae* cultures were streaked on sheep blood agar and incubated at 30°C for 24–48 h. Haemolytic patterns were examined and categorised into α-haemolysis (grass green translucent haemolysis ring around colonies), β-haemolysis (wide transparent haemolysis ring around colonies) and γ-haemolysis (no haemolysis ring around colonies). *S. aureus* ATCC25923, exhibiting β-haemolysis, served as positive control.

#### 2.13.2 Tolerance of BTS1-KO *S. cerevisiae* to simulated gastrointestinal fluids

Pepsin was prepared at a concentration of 3 mg/mL in PBS and adjusted to pH 3.0 with hydrochloric acid, which was filtered using a syringe filter (aperture 0.22 μm) for reserve. Trypsin was prepared at 1 mg/mL by PBS, supplemented with 0.3% of bovine bile salt and adjusted to pH 8.0 with sodium hydroxide, which was filtered using a syringe filter (pore size 0.22 μm) for later use. BTS1-KO *S. cerevisiae* was inoculated in YPD broth and cultured at 30°C and 200 rpm for 16 h. After culturing, the cells were collected by centrifugation at 4000×g for 12 min at 4°C and washed twice with PBS. Then, the bacteria were resuspended in the prepared artificial gastric juice and intestinal juice, respectively. Viable bacteria counts were determined after 1 h and 3 h of incubation in artificial gastric juice and after 2 h and 4 h in intestinal juice.

#### 2.13.3 Auto-aggregation and co-aggregation assay

BTS1-KO *S. cerevisiae* and BY4743 were cultured at 30°C for 16 h, then centrifuged at 8000 ×g at 4°C for 10 min to collect bacteria. The bacterial pellets were washed twice with PBS and resuspended in PBS, and the absorbance (A_0_) was measured at 600 nm. *S. cerevisiae* BY4743 served as the control. The bacterial suspensions were left at room temperature for 5 h, and the absorbance was measured again (A_t_). Auto aggregation (%) was calculated using the following equation:


$$\text{Auto aggregation }\left(\%\right)=\left[1-\left({\text{A}}_{\text{t}}/{\text{A}}_{0}\right)\right]\times 100$$

For co-aggregation, bacterial suspensions of BTS1-KO *S. cerevisiae* and BY4743 were prepared as described above. Overnight cultures of *E. coli* and *S. aureus* in LB broth medium were centrifuged at 8000 ×g at 4°C for 10 min to obtain precipitate, which was then washed with PBS and resuspended to obtain bacterial suspensions. *S. cerevisiae* BY4743 served as the control. The absorbance of *S. cerevisiae* suspension (Ａ_probio_) and bacterial suspension (A _pat_) was measured at 600 nm. Equal volumes (2 mL) of *S. cerevisiae* and bacterial suspensions were mixed, and incubated at 37°C for 5 h, and the absorbance of the mixed solution (A _mix_) was measured. The Co-aggregation (%) was calculated using the equation:


$$\begin{array}{c} \text{Co-aggregation }\left(\%\right)\\ =[(\left({\text{A}}_{\text{probio}}+{\text{A}}_{\text{pat}}\right)/2-{\text{A}}_{\text{mix}})/\left({\text{A}}_{\text{probio}}+{\text{A}}_{\text{pat}}\right)/2]\times 100 \end{array}$$

### 2.14 Establishment of *Galleria mellonella* infection model

Precipitated BTS1-KO *S. cerevisiae* cultured at 30°C for 16 h was washed with PBS three times and resuspended in PBS (Alive *S. cerevisiae*). For heat-inactivated probiotic suspension, a portion (10 mL) of the above suspension was autoclaved at 121°C for 15 min, then centrifuged at 8000×g for 10 min, washed and resuspended with PBS (Heat-inactivated *S. cerevisiae*). The *G. mellonella* larvae were randomly divided into eight groups: one blank control group (PBS group); one control group (Pathogenic bacteria group); three toxicity test groups (CFS group, Alive group and Heat-inactivated group); three treatment groups (CFS + Pathogenic bacteria, Alive + Pathogenic bacteria, Heat-inactivated + Pathogenic bacteria).

### 2.15 Determination of antibacterial activity of farnesene

The antibacterial activity of farnesene was assessed using a standardised broth microdilution method following the Clinical and Laboratory Standards Institute (CLSI) guidelines (M27-A3) (CLSI, 2008). The inhibitory activity of farnesene against pathogenic bacteria was tested in 96-well plates.

### 2.16 Analysis of organic acids in CFS using ultra-performance liquid chromatography-tandem mass spectrometry

Organic acid types and concentrations in CFS were analysed using a Waters Acquity I class UPLC coupled to a Waters XEVO TQD mass spectrometer. The CFS of BTS1-KO *S. cerevisiae*, CFS of BY4743 and YPD medium were thawed at 4°C. Then, 5 μL of the supernatant of the sample to be tested was taken into a centrifuge tube, and 35 μL of the configured derivatisation reagent (4.8 μL of AQB and 2 μL of DIPEA dissolved in 2 mL of acetonitrile) and 35 μL of the condensation agent (15.2 mg of HATU dissolved in 2 mL of acetonitrile) were added. After vortexing for 20 min, 100 μL of PBS and 300 μL of methyl tert-butyl ether were added, vortexed for 10 min, and then centrifuged at 6000 rpm at 4°C for 5 min. A total of 50 μL of supernatant was collected in a new centrifuge tube and concentrated by centrifugation at 40°C for 20 min in a vacuum concentrator. The sample was then dried and reconstituted by adding 100 μL of 10% acetonitrile in water; After vortexing for 5 min and centrifuging at 12000 rpm for 15 min at 4°C, 40 μL of supernatant was transferred to the injection vial, and the type and concentration of organic acids were analysed using UPLC-MS/MS.

### 2.17 Statistical analysis

All experiments were set up in three replicate groups and repeated independently three times. Statistical analysis and plotting were performed using GraphPad Prism software. Experimental data were statistically tested using Student’s *t*-test or one-way ANOVA. Statistical differences were determined based on *P* values of *P<0.05, **P<0.01, and ***P<0.005.

## 3 Results

### 3.1 Antibacterial effect by CFS of BTS1-KO *S. cerevisiae*

**Figure 1:**
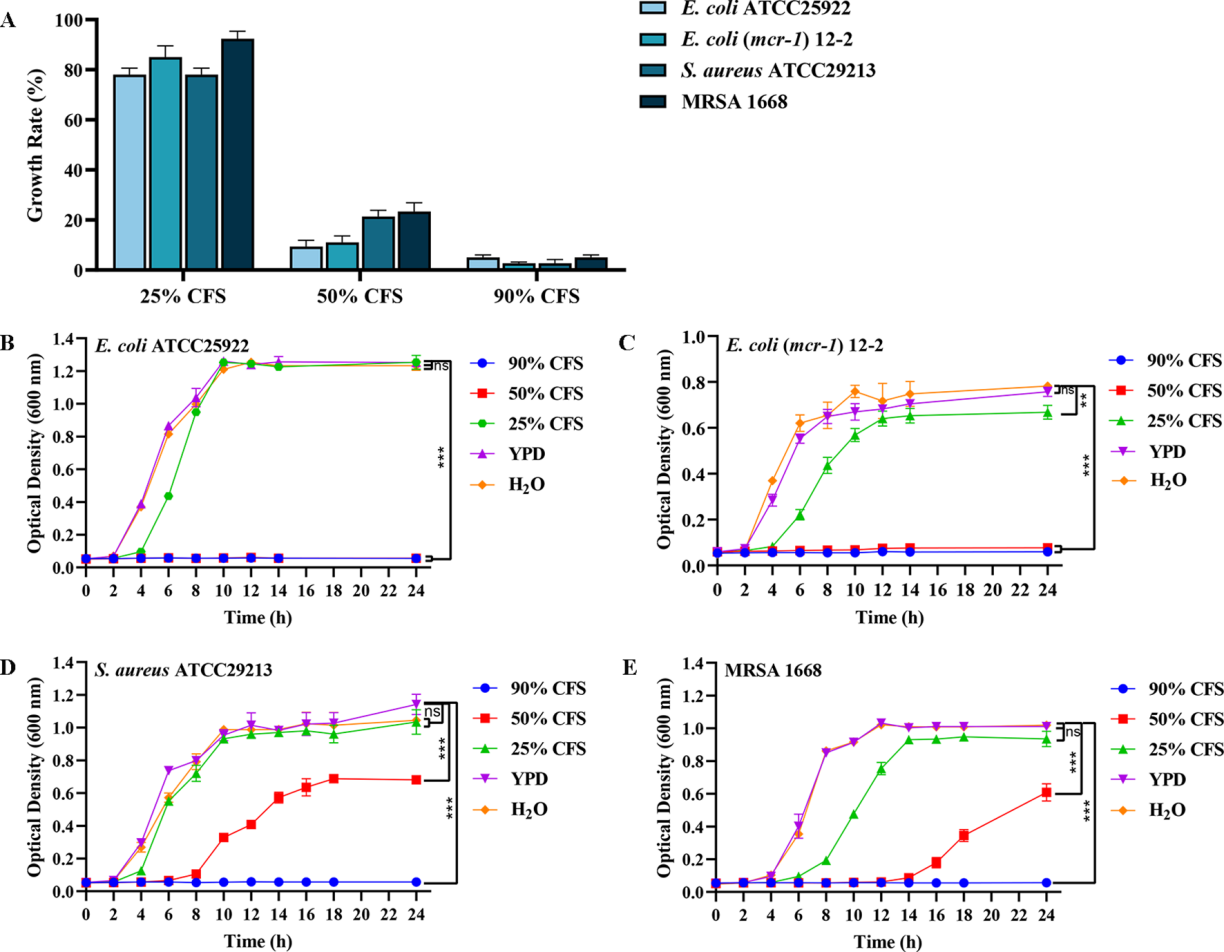
Determination of antibacterial activity of cell-free supernatant (CFS) of BTS1-KO *S. cerevisiae*. **(A)** Minimum inhibitory concentration (MIC)value of CFS against drug-sensitive and drug-resistant strains of *E*. *coli* ATCC25922, *E*. *coli* (*mcr*-*1*) 12-2, *S. aureus* ATCC29213 and MRSA 1668; **(B)** Growth curve of *E*. *coli* ATCC25922 after CFS treatment; **(C)** Growth curve of *E*. *coli* (*mcr*-*1*) 12-2 after CFS treatment; **(D)** Growth curve of *S. aureus* ATCC29213 after CFS treatment; **(E)** Growth curve of MRSA 1668 after CFS treatment. Data are representative of three independent experiments and presented as mean ± SD. ns indicates no statistical significance; **P < 0.01; ***P < 0.001.

Screening of 1800 *S. cerevisiae* mutants, BTS1-KO *S. cerevisiae* exhibited potent antibacterial activity. The MIC_80_ of CFS against *E. coli* ATCC25922, *E. coli* -mcr1 12-2, *S. aureus* ATCC29213 and MRSA 1668 was 90%, 50%, 50% and 50%, respectively, compared to the control group (**Figure 1A**). As the concentration of CFS decreased, the antibacterial activity gradually diminished. Furthermore, BTS1-KO *S. cerevisiae* demonstrated effectiveness in inhibiting the proliferation of various bacteria, including gram-negative, gram-positive and drug-resistant bacteria. Specifically, CFS inhibited *E. coli* (*mcr*-*1*) 12-2 and *S. aureus* ATCC29213 by 98%; *S. aureus* ATCC25923 and *S.* Typhimurium SL1344 by 96%; *E. coli* ATCC25922, MRSA 1668, *P. aeruginosa* ATCC27853 and *K. pneumoniae* ATCC1705 by 95%; *P. aeruginosa* 1554 by 94%; *K. pneumoniae* ATCC700603, *K. pneumoniae* ATCC1706 and *A. baumannii* 21-1 by 93%; and *K. pneumoniae* 2118 by 92%. Importantly, BTS1-KO *S. cerevisiae* exhibited comparable activity to well-known probiotics like *L. rhamnosus* and surpassed *S. boulardii* (**Table 1**). These results underscored the potent antibacterial activity of BTS1-KO *S. cerevisiae* CFS.

**Table 1:** Antimicrobial of CFS against different bacteria.

Stains ^a^	Growth rate		
	BTS1-KO	*S. boulardii*	*L. johnsonii*
*E. coli* ATCC25922	4.80% ± 0.72%	10.21% ± 0.93% *	7.76% ± 0.81% ^ns^
*E. coli*(*mcr-1*)12-2	2.95% ± 0.94%	18.86% ± 4.05% ***	10.45% ± 1.07% ^ns^
*S. aureus* ATCC29213	2.82% ± 0.33%	14.97% ± 1.89% ***	6.23% ± 1.24% ^ns^
*S. aureus* ATCC25923	4.93% ± 0.73%	26.24% ± 2.83% ***	6.40% ± 0.94% ^ns^
MRSA 1668	5.56% ± 0.65%	33.61% ± 1.61% ***	6.87% ± 1.59% ^ns^
*P. aeruginosa* ATCC27853	5.34% ± 0.20%	29.68% ± 4.85% ***	10.78% ± 0.55% *
*P. aeruginosa* 1554	9.17% ± 0.32%	15.31% ± 5.10% **	5.28% ± 0.66% ^ns^
*K. pneumoniae* ATCC700603	9.06% ± 0.72%	36.51% ± 1.24% ***	7.27% ± 2.04% ^ns^
*K. pneumoniae* ATCC1706	7.28% ± 0.21%	32.40% ± 4.66% ***	5.33% ± 0.54% ^ns^
*K. pneumoniae* ATCC1705	6.02% ± 0.86%	37.98% ± 1.72% ***	9.97% ± 1.83% ^ns^
*K. pneumoniae* 2118	9.27% ± 1.01%	34.55% ± 1.97% ***	9.14% ± 0.91% ^ns^
*A. baumannii* 21-1	8.64% ± 0.66%	28.05% ± 5.45% ***	4.47% ± 0.66% ^ns^
*S.* Typhimurium SL1344	4.43% ± 0.57%	21.92% ± 8.18% ***	6.08% ± 1.51% ^ns^

^ns^indicates no statistical significance; *P < 0.05; ***P < 0.001.

a *E. coli* ATCC25922 is the standard strain of *E. coli*; *E. coli*(*mcr-1*)12-2 is a clinical isolate of *E. coli* carrying polymyxin resistance gene (*mcr*-*1*); *S. aureus* ATCC29213 and *S. aureus* ATCC25923 are *S. aureus* standard strains; MRSA 1668 is a clinical isolate of methicillin-resistant *S. aureus*; *P. aeruginosa* ATCC27853 is the *P. aeruginosa* standard strain; *P. aeruginosa* 1554 is a clinically isolated drug-resistant strain of *P. aeruginosa*; *K. pneumoniae* ATCC700603, *K. pneumoniae* ATCC1706 and *K. pneumoniae* ATCC1705 are *K. pneumoniae* standard strains; *K. pneumoniae* 2118 is a clinically isolated drug-resistant strain of *K. pneumoniae*; *A. baumannii* 21-1 is a clinically isolated drug-resistant strain of *A. baumannii*; *S.* Typhimurium SL1344 is a *S.* Typhimurium carrying streptomycin resistance. *S. boulardii* and *L. johnsonii* are controls.

### 3.2 Growth inhibition effect of BTS1-KO *S. cerevisiae* treatment on *E. coli* and *S. aureus*

The growth curve analysis of *E. coli* and *S. aureus* treated with BTS1-KO *S. cerevisiae* CFS revealed significant inhibition compared to controls. H_2_O was used instead of YPD broth to examine the effect of nutritional differences on the experimental group. Differences in bacterial growth between the blank control (representing the minimum amount of additional nutrients) and the YPD broth control (representing the maximum amount of additional nutrients) were not significant, suggesting that the bacterial cultures were nutrient-rich and leading to the hypothesis that nutrient depletion did not contribute to the changes in growth. When treated with 90% and 50% CFS, the growth of *E. coli* ATCC25922 was significantly inhibited, with no significant change in bacterial absorbance within 24 h (**Figure 1B**). Similarly, treatment with 25% CFS reduced the growth rate of *E. coli* (*mcr*-*1*) 12-2, with a lag period from 2 to 4 h, and the total bacterial content at 24 h reduced to approximately 85% of the control. Notably, 90% and 50% CFS effectively inhibited the growth of *E. coli* (*mcr*-*1*) 12-2 within 24 h (**Figure 1C**). Treatment with 50% CFS significantly altered the growth curve of *S. aureus* ATCC29213, delaying the logarithmic growth phase until 10 h, with the total bacterial content at 24 h reduced to about 53% of the control (**Figure 1D**). Similarly, MRSA growth was significantly delayed, entering the logarithmic growth phase only after 16 h of growth in the presence of 50% CFS, while hardly growing in the presence of 90% CFS throughout the growth phase (**Figure 1E**). These findings indicated that BTS1-KO *S. cerevisiae* CFS effectively inhibited the growth of both *E. coli* and *S. aureus*, with the inhibitory effect showing dose dependence.

### 3.3 The inhibitory effect of CFS of BTS1-KO *S. cerevisiae* on the biofilm formation

**Figure 2:**
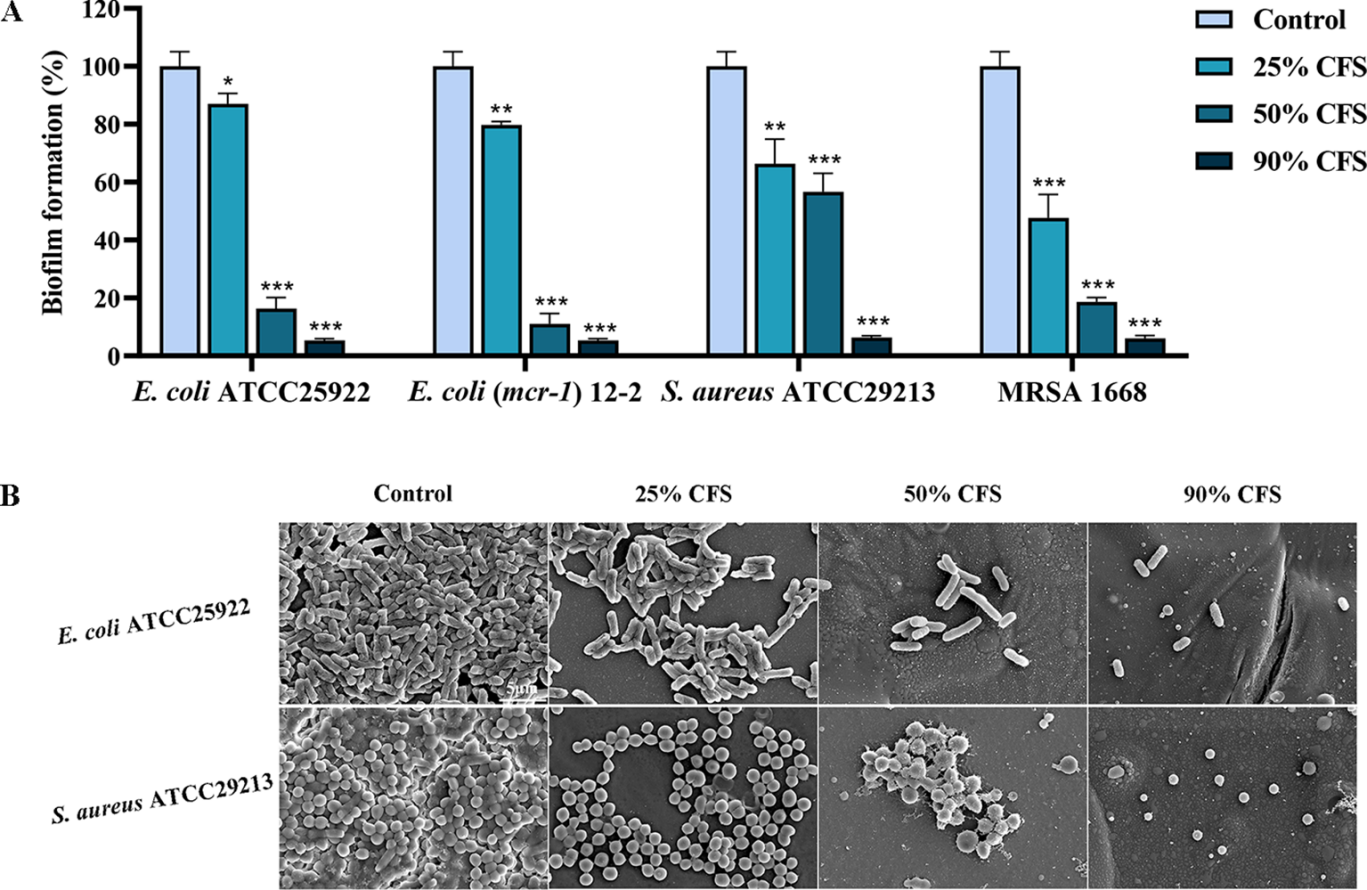
Effect of cell-free supernatant (CFS) of BTS1-KO *S. cerevisiae* on the biofilm of pathogenic bacteria (*E. coli* ATCC25922. *E*. *coli (mcr-1) 12-2*, *S. aureus* ATCC29213, MRSA) in 96-well plates. Pathogenic bacteria treated with CFS were incubated in LB medium at 37°C for 24 h. After incubation, a crystal violet assay **(A)**, microscopy and SEM **(B)** were used to assess biofilm formation. The bars in B represent 5 μm. Results in A are presented as means ± SDs. ns indicates no statistical significance; *P < 0.05; **P < 0.01; ***P < 0.001.

Biofilm formation confers strong resistance to pathogenic bacteria (Roy et al., 2018). The inhibitory effect of BTS1-KO *S. cerevisiae* CFS on *E. coli* and *S. aureus* biofilm formation was quantitatively assessed using the CV reduction assay. Results demonstrated that CFS effectively inhibited the biofilm formation of *E. coli* and *S. aureus* in a dose-dependent manner. Specifically, treatment with 25% CFS significantly inhibited biofilm formation. Under 90% CFS treatment, the inhibition rates of *E. coli* ATCC25922, *E. coli* (*mcr*-*1*) 12-2, *S. aureus* ATCC29213 and MRSA 1668 were 94.7%, 94.7%, 94.0%, and 93.7%, respectively(**Figure 2A**). Additionally, SEM images of biofilms revealed a significant reduction in bacterial density and more dispersed arrangements in CFS-treated groups compared to untreated groups, which showed a higher bacterial density and tight arrangement (**Figure 2B**). These findings underscored the ability of BTS1-KO *S. cerevisiae* CFS to effectively inhibit biofilm formation in both *E. coli* and *S. aureus*.

### 3.4 CFS reduces adhesion of *E. coli* and *S. aureus*

**Figure 3:**
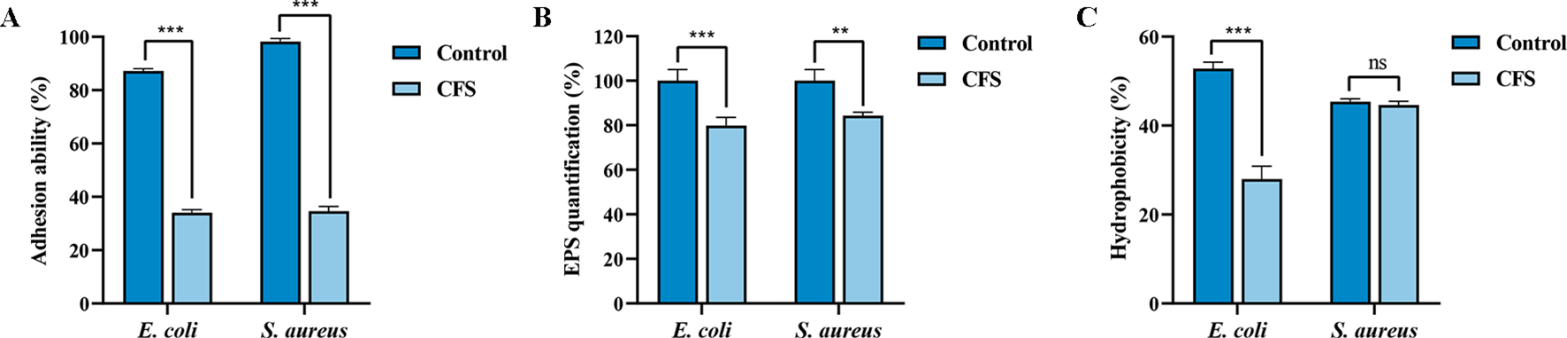
Effects of cell-free supernatant (CFS) of BTS1-KO S. cerevisiae on adhesion ability, EPS production and hydrophobicity of *E. coli* ATCC25922 and *S. aureus* ATCC29213. **(A)** CFS reduced the adhesion of *E. coli* and *S. aureus*; **(B)** CFS reduced EPS production of *E. coli* and *S. aureus*; **(C)** CFS reduced hydrophobicity of *E. coli* and *S. aureus*. Results are presented as means ± SDs. ns indicates no statistical significance; **P < 0.01; ***P < 0.001.

Adhesion is the initial step in biofilm formation, facilitated by the secretion of extracellular polymer matrix, such as EPS produced by pathogens., which in turn can be used to resist antibiotics and other compounds (Lourenço et al., 2012). The results showed that the adhesion abilities of *E. coli* and *S. aureus* were reduced by 53.24% and 63.56%, respectively, in the 50% CFS-treated group compared to the CFS-untreated group (**Figure 3A**). Moreover, EPS production by *E. coli* and *S. aureus* decreased by 20.18% and 15.69%, respectively, after CFS treatment (**Figure 3B**), indicating a reduction in adhesion ability and consequently in biofilm formation. Additionally, surface hydrophobicity, indirectly reflecting adhesion ability (Yang et al., 2022), was reduced by 18.09% in CFS-treated *E. coli*, although no significant effect was observed in *S. aureus* (**Figure 3C**).

### 3.5 BTS1-KO *S. cerevisiae* can directly inhibit the growth of *E. coli* and *S. aureus*

**Figure 4:**
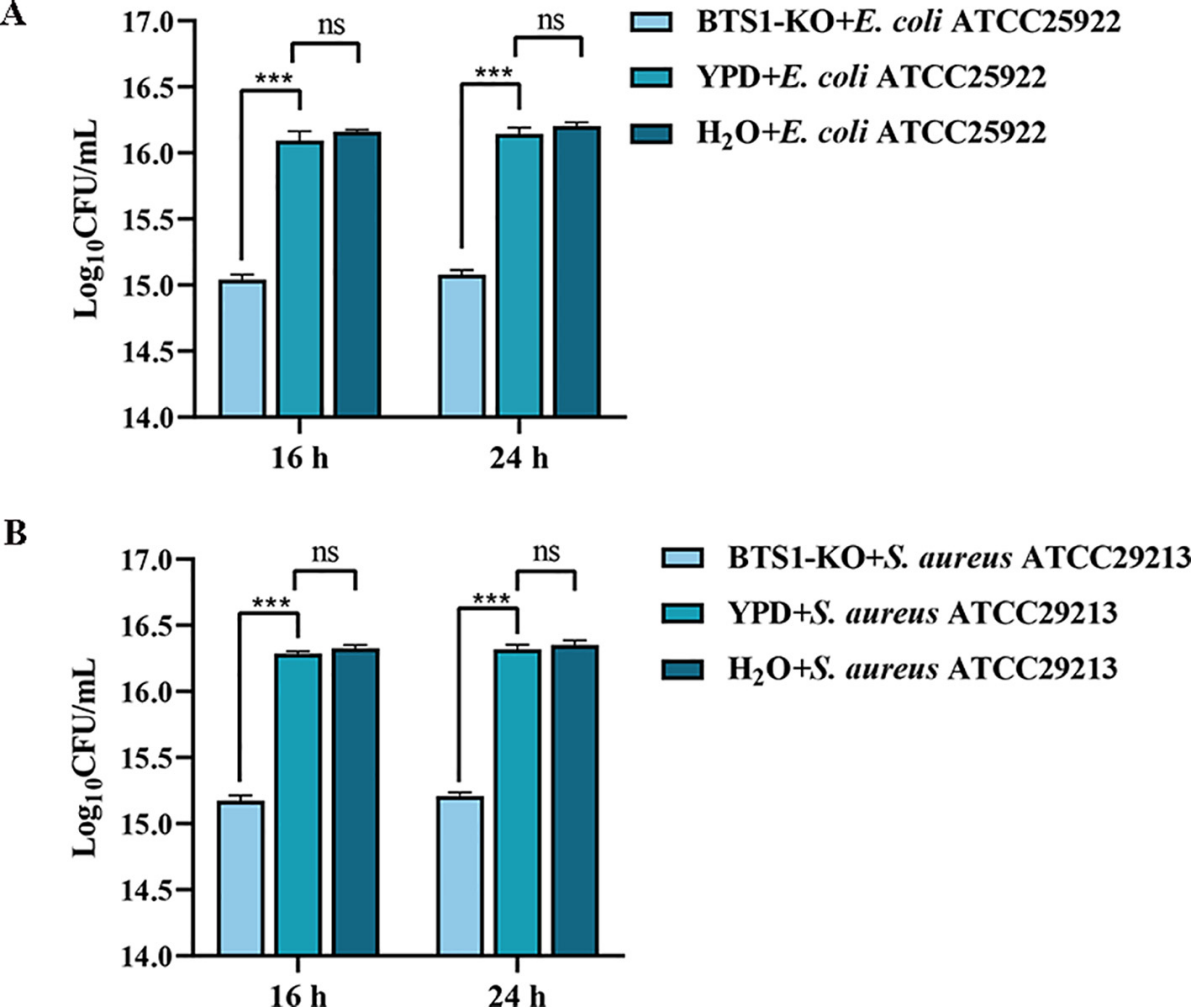
Co-culture of BTS1-KO *S. cerevisiae* and pathogenic bacteria. **(A)** Survival number of *E*. *coli* cultured alone or co-cultured with BTS1-KO *S. cerevisiae*; **(B)** Survival number of *S. aureus* cultured alone or co-cultured with BTS1-KO *S. cerevisiae*. Results are presented as means ± SDs. ns indicates no statistical significance; ***P < 0.001.

Using the bacteria-*S. cerevisiae* co-culture model, we verified the ability of live BTS1-KO *S. cerevisiae* to inhibit the growth of pathogenic bacteria. Results indicated a significant reduction in *E. coli* cell numbers after co-culture with live BTS1-KO *S. cerevisiae* compared to YPD medium and H_2_O controls. At 16 and 24 h, *E. coli* growth inhibition rates were 91.20% (1.1×10^15^ CFU/mL) and 91.43% (1.2×10^15^ CFU/mL), respectively (**Figure 4A**). Moreover, a significantly lower cell number of the *S. aureus* co-culture group was determined compared to the single culture groups, and inhibition rates of 80.43% (1.5×10^15^ CFU/mL) and 85.16% (1.58×10^15^ CFU/mL) were reported at 16 h and 24 h, respectively (**Figure 4B**). Additionally, YPD broth was replaced with sterile water as a blank control group. The indicator bacteria culture was revealed to be nutrient-rich, with nutrient depletion not contributing to growth changes. These results confirmed the inhibitory effect of BTS1-KO *S. cerevisiae* cells on the growth of both *E. coli* and *S. aureus*.

### 3.6 BTS1-KO *S. cerevisiae* had good probiotic properties

#### 3.6.1 BTS1-KO *S. cerevisiae* possessed no haemolytic activity

**Figure 5:**
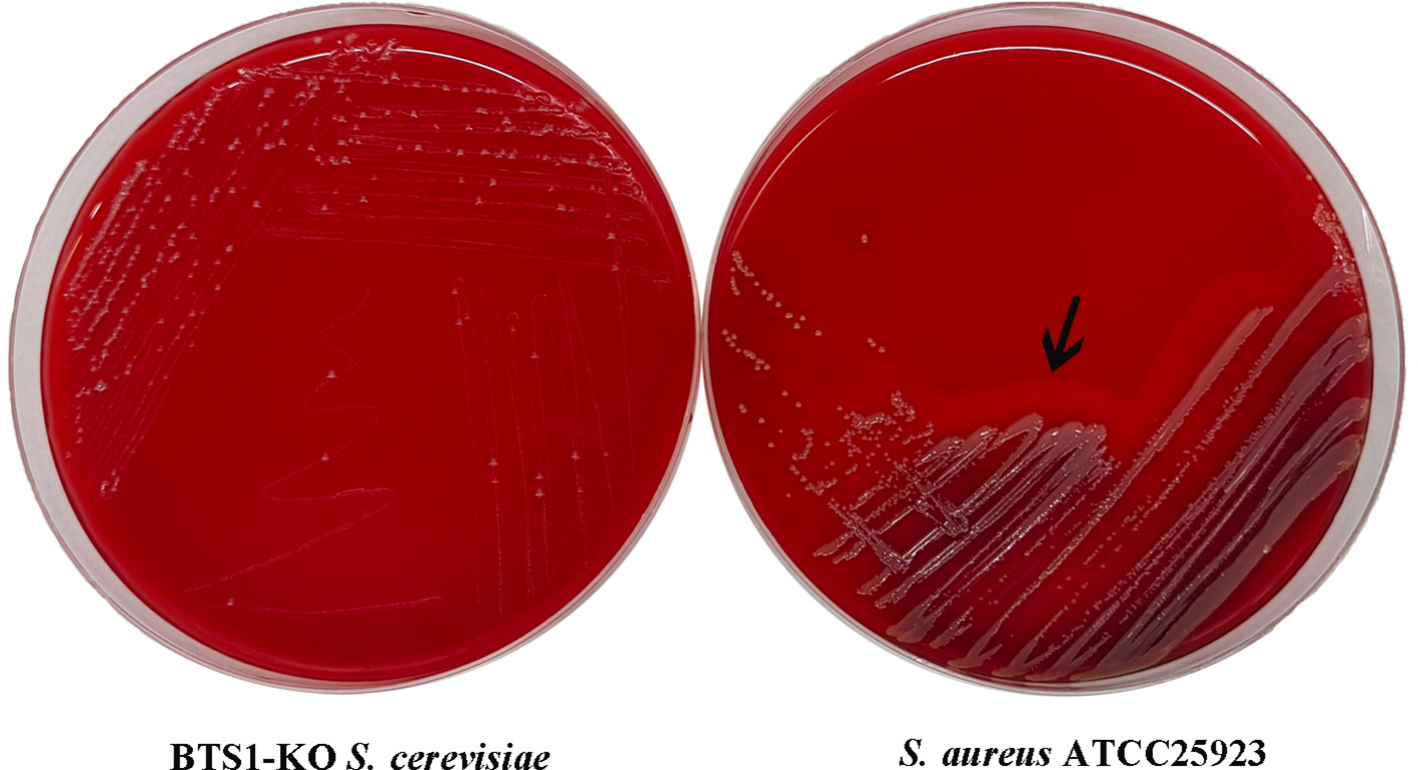
BTS1-KO *S. cerevisiae* possessed no hemolytic activity. To assess the safety of BTS1-KO *S. cerevisiae*, hemolytic activity was tested on sheep blood agar, with *S. aureus* ATCC25923 as a control. *S. aureus* ATCC25923 displayed a clear hemolytic ring (β-hemolysis) (right), while BTS1-KO *S. cerevisiae* showed no hemolytic activity (left).

To evaluate the cytotoxicity of BTS1-KO *S. cerevisiae*, we assessed its haemolytic activity against erythrocytes. Unlike the β-haemolytic activity observed with the reference strain *S. aureus* ATCC25923 (**Figure 5**), BTS1-KO *S. cerevisiae* did not induce haemolysis when grown on sheep blood agar. Consequently, BTS1-KO *S. cerevisiae* was considered safe for human application.

#### 3.6.2 Tolerance analysis of artificially simulated gastric and intestinal fluid

**Figure 6:**
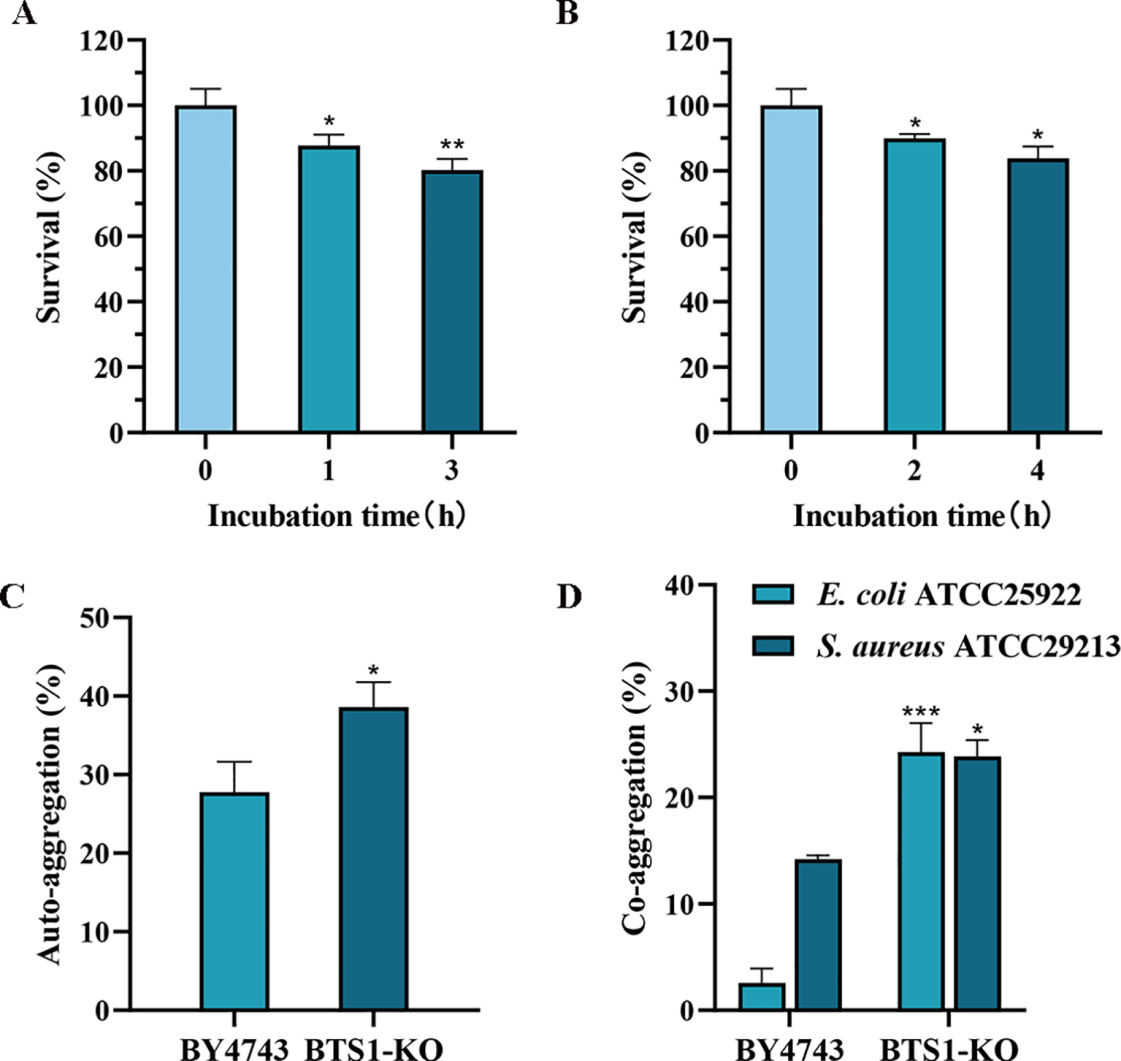
Evaluation of Probiotic Properties. **(A)** Tolerance to simulated gastric fluid. The survival rate of BTS1-KO *S. cerevisiae* after incubation in simulated gastric fluid with pH 3.0 for 1 h and 3 h; **(B)** Tolerance to 0.3% bile salts. The survival rate of BTS1-KO *S. cerevisiae* after 2 h and 4 h incubation in artificial intestinal fluid containing 0.3% bile salt; **(C)** The auto-aggregation ability of BTS1-KO *S. cerevisiae* and *S. cerevisiae* BY4743, with the auto-aggregation ability of BTS1-KO *S. cerevisiae* showing a significant increase; **(D)** The co-aggregation ability of BTS1-KO *S. cerevisiae* and *S. cerevisiae* BY4743 with pathogenic bacteria, with BTS1-KO S. cerevisiae showing a significantly increased co-aggregation ability. Results are shown as means ± SDs. *P < 0.05; **P < 0.01; ***P < 0.001.

To colonise the gastrointestinal tract and play a beneficial role, probiotics must possess a certain tolerance to the digestive tract environment. Therefore, we detected the tolerance of BTS1-KO *S. cerevisiae* to artificially simulated gastric and intestinal fluids. After incubation in simulated gastric fluid with pH 3.0 for 1 h, the survival rate of BTS1-KO *S. cerevisiae* was as high as 87%. After 3 h of incubation, the survival rate remained higher than 80% (**Figure 6A**). Additionally, in the artificial intestinal fluid, survival rates were 89.93% and 83.87% after 2 h and 4 h of incubation, respectively (**Figure 6B**). These findings demonstrated the robust tolerance of BTS1-KO *S. cerevisiae* to gastrointestinal fluids.

#### 3.6.3 The auto aggregation and co-aggregation abilities with pathogenic bacteria and BTS1-KO *S. cerevisiae*

Auto aggregation and co-aggregation abilities are indicative of the potential for colonisation in the human intestinal tract (Vlková et al., 2008). Compared to *S. cerevisiae* BY4743, BTS1-KO *S. cerevisiae* exhibited improved auto aggregation, increasing from 27.75% to 38.58% (**Figure 6C**). Moreover, compared with *E. coli* and *S. aureus*, the co-aggregation ability of BTS1-KO *S. cerevisiae* increased by 21.69% and 9.65%, respectively (**Figure 6D**), indicating its capacity for close contact with *E. coli* and *S. aureus* and exert enhanced antibacterial effects.

### 3.7 Antibacterial effect of BTS1-KO *S. cerevisiae in vivo*

**Figure 7:**
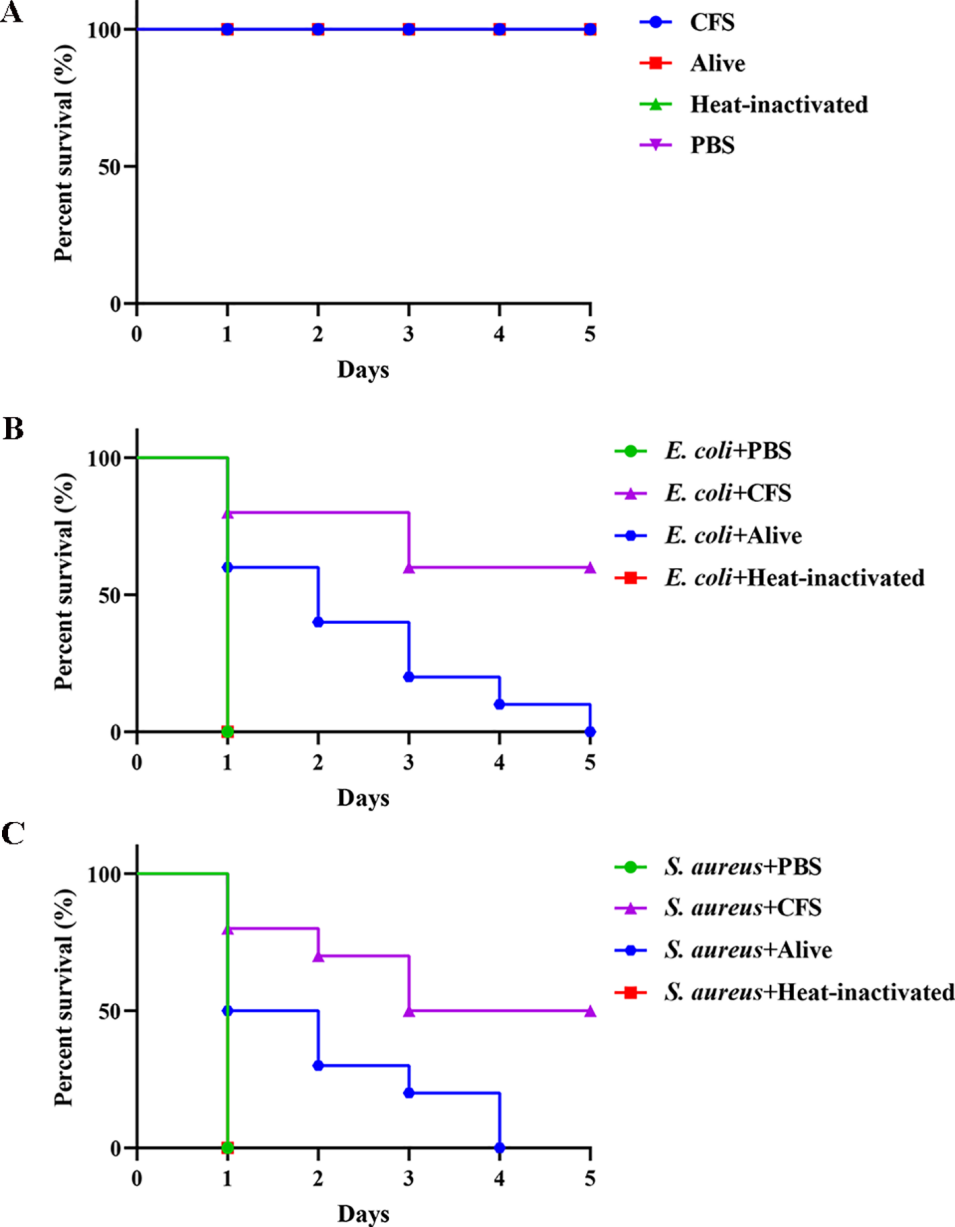
The effect of BTS1-KO *S. cerevisiae* on the *G*. *mellonella*- pathogenic bacteria infection model. **(A)** Healthy larvae were incubated with CFS, alive BTS1-KO *S. cerevisiae*, heat-inactivated BTS1-KO *S. cerevisiae* and PBS for 5 days, respectively. PBS was the blank control; **(B)** *E*. *coli*-infected larvae were treated with PBS (control), CFS, live BTS1-KO *S. cerevisiae* or heat-inactivated BTS1-KO *S. cerevisiae*. Larvae were monitored daily, and survival rates were calculated; **(C)** *S. aureus*-infected larvae were treated with PBS (control), CFS, live BTS1-KO *S. cerevisiae* or heat-inactivated BTS1-KO *S. cerevisiae*. Larvae were monitored daily, and survival rates were calculated.

The *G. mellonella* larvae model, resembling mammalian systems, was employed to evaluate the *in vivo* antibacterial activity of BTS1-KO *S. cerevisiae* (Ménard et al., 2021). Compared with the PBS control group, a single injection of live *S. cerevisiae*, heat-inactivated *S. cerevisiae* and CFS had no significant effect on the survival of healthy larvae (**Figure 7A**). According to the survival curve, the survival time of larvae infected with *E. coli* or *S. aureus* in the CFS or live cells treated groups was greater than the control group. In the *E. coli*-infected larvae model, larvae in the CFS-treated group survived longer than those in the control group, with a survival rate of 60% (**Figure 7B**). The survival time of the infected *S. aureus* larvae treated with CFS was also greater than the control groups, with a survival rate of 50% (**Figure 7C**). Additionally, live BTS1-KO *S. cerevisiae* prolonged the survival time of *E. coli*-infected *G. mellonella*, with a survival rate of 60% observed on the first day (**Figure 7B**). Live BTS1-KO *S. cerevisiae* also prolonged the survival time of *S. aureus*-infected *G. mellonella*, which was infected for 4 days before all died (**Figure 7C**). However, heat-inactivated BTS1-KO *S. cerevisiae* showed no therapeutic effect on *G. mellonella*, suggesting that live BTS1-KO *S. cerevisiae* is required for effective protection *in vivo* (**Figures 7B, C**). These results underscored the potential of BTS1-KO *S. cerevisiae* as a therapeutic agent against pathogenic bacterial infections *in vivo*.

### 3.8 Antibacterial effect of farnesene

**Figure 8:**
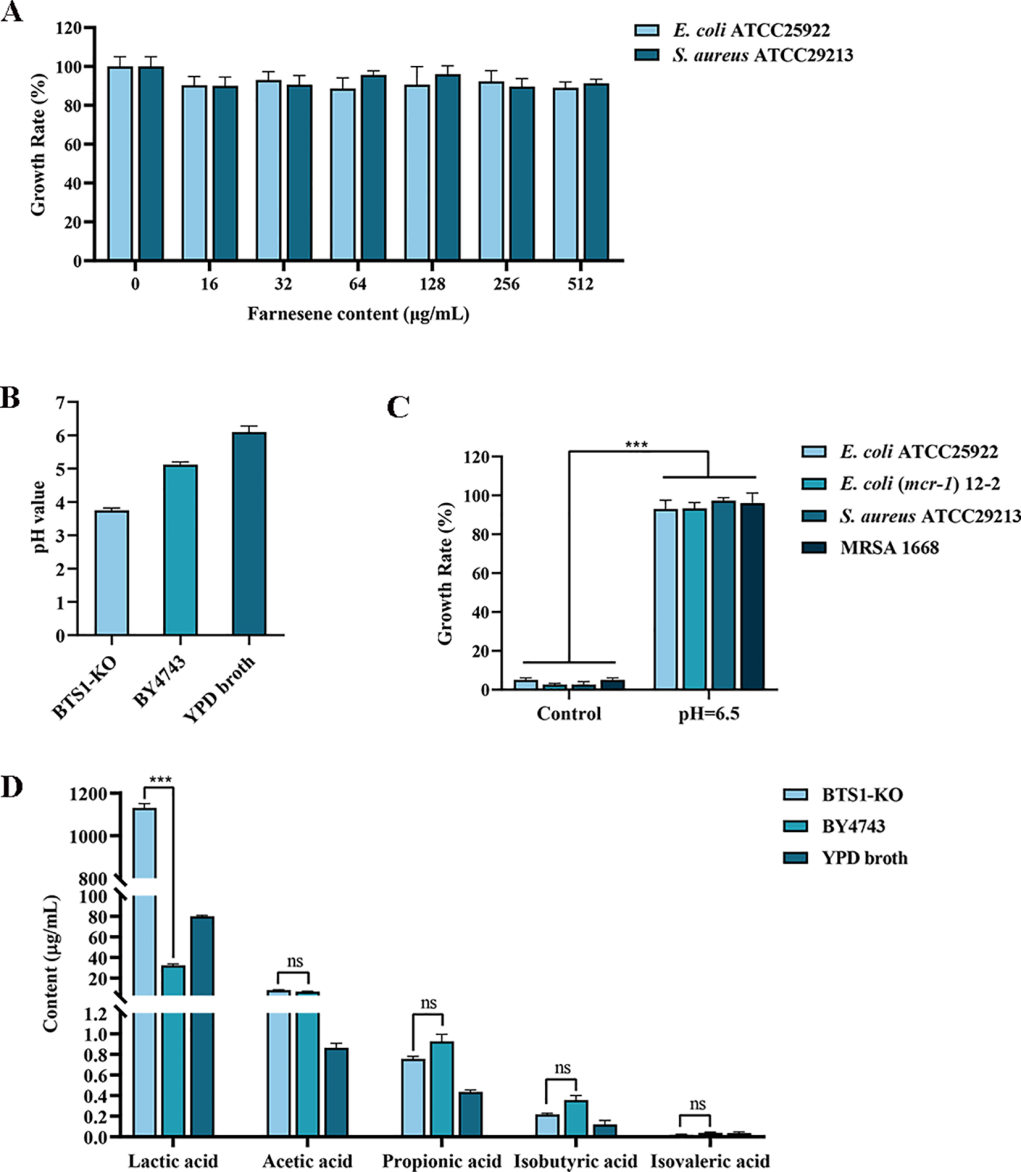
Metabolites produced by BTS1-KO *S. cerevisiae*. **(A)** Determination of the antibacterial effect of farnesene; **(B)** The pH value of CFS of BTS1-KO *S. cerevisiae*, CFS of *S. cerevisiae* BY4743 and YPD medium; **(C)** When the pH of CFS was adjusted to 6.5, its antibacterial activity disappeared; **(D)** The type and content of organic acids in the CFS of BTS1-KO *S. cerevisiae*, CFS of *S. cerevisiae* BY4743 and YPD medium. Results are presented as means ± SDs. ns indicates no statistical significance; ***P < 0.001.

*BTS1* encodes geranylgeranyl diphosphate synthase (GGPPS) gene, an enzyme involved in terpenoid biosynthesis (Jiang et al., 1995). Experiments have confirmed that the content of farnesene increased after *BTS1* was knocked out (Wang et al., 2022). In this study, the antibacterial activity of farnesene was assessed, revealing that farnesene exerted no antibacterial activity, which indicated that increased farnesene levels were not responsible for the observed antibacterial effects of BTS1-KO *S. cerevisiae* (**Figure 8A**).

### 3.9 Analysis of organic acids produced by BTS1-KO *S. cerevisiae*

*S. boulardii* produces organic acids such as lactic acid, acetic acid and formic acid to exert its antibacterial activity (Pais et al., 2020). Therefore, we determined the pH of the CFS of BTS1-KO *S. cerevisiae* and CFS of BY4743, which were 3.75 and 5.12, respectively (**Figure 8B**). Then, 2 mol/L NaOH solution was used to adjust the pH of CFS to 6.5, which resulted in the loss of the antibacterial activity of CFS (**Figure 8C**). UPLC-MS/MS analysis revealed a significantly higher content of lactic acid in BTS1-KO *S. cerevisiae* CFS (35-fold increase) compared to *S. cerevisiae* BY4743, while the content of acetic acid, propionic acid, isobutyric acid and isovaleric acid were not significant, suggesting lactic acid production as a major contributor to its antimicrobial activity (**Figure 8D**). Thus, increased lactic acid production and the consequent decrease in pH likely contribute to the antimicrobial effects of BTS1-KO *S. cerevisiae*.

## 4 Discussion

Human health is significantly impacted by various pathogenic bacteria, with some high-incidence pathogens like *S. aureus*, *E. coli* and *K. pneumoniae* posing substantial threats (Toone, 2011). For example, in 2019, bacterial infections led to 7.7 million deaths, with 1.1 million deaths associated with *S. aureus*, 950,000 with *E. coli* and 790,000 with *K. pneumoniae* (Lancet, 2022). Currently, clinical bacterial infections are often caused by multiple bacteria, and the misuse of antibiotics has further exacerbated the problem by promoting the emergence of drug-resistant bacteria and superbugs. Additionally, biofilm formation increases their drug resistance and pathogenicity, underscoring the need to search for new antimicrobial strategies (Ferri et al., 2017).

Probiotics have been recognised as an effective anti-infection strategy. In this study, the BTS1-KO *S. cerevisiae* strain effectively exerted antibacterial effects against most clinically isolated bacterial pathogens, comparable to the well-known probiotics *S. boulardii* and *L. johnsonii*. Importantly, it exhibited efficacy against multidrug-resistant strains and showed no toxicity to *G. mellonella*. Haemolysis experiment also revealed that BTS1-KO *S. cerevisiae* had no haemolytic activity, indicating that it was non-pathogenic to human and had high safety. Furthermore, to confirm the therapeutic efficacy of BTS1-KO *S. cerevisiae in vivo*, we utilised the *G. mellonella* infection model. The model is widely used to assess the virulence of a wide range of pathogenic bacteria, including *E. coli* and *S. aureus*, and antibacterial drug efficiency (Mikulak et al., 2018). The results revealed that BTS1-KO *S. cerevisiae* prolonged the survival time of the larvae infected with *E. coli* and *S. aureus*.

The inhibitory effect observed in co-culture experiments was attributed to the high auto aggregation and high co-aggregation abilities of BTS1-KO *S. cerevisiae* with pathogenic bacteria. The high auto aggregation ability of BTS1-KO *S. cerevisiae* enhanced its adhesion ability, prompted its close contact with intestinal mucosa *in vivo*, prolonged its residence time in the intestines and enabled effective exertion of its antibacterial activity (Rodríguez Arreola et al., 2021). Moreover, the high co-aggregation ability of BTS1-KO *S. cerevisiae* with *E. coli* and *S. aureus* forms a barrier, preventing colonisation and enabling the release of anti-pathogen substances (Ocaña and Nader-Macías, 2002). For example, *Lactobacillus rhamnosus* has been reported to possess good adhesion properties, as evidenced by its prevention of the internalisation of enterohaemorrhagic *E. coli* in human enterocyte cell lines (Huang et al., 2023). These findings suggest that BTS1-KO *S. cerevisiae* competes with other bacteria for the binding sites *in vivo*, thereby inhibiting bacterial colonisation and proliferation and reducing their pathogenicity.

When ingesting probiotics orally, they have to withstand the effects of stomach acid and bile salts before reaching the interior of the intestines and adhering to the epithelial cells. After arriving at the intestines, probiotics inhibit the proliferation of pathogenic bacteria and form an antimicrobial biological barrier (Jiang et al., 2023). Moreover, the survival of BTS1-KO *S. cerevisiae in vitro* under simulated gastrointestinal conditions was assessed to predict its actual survival *in vivo* when consumed in a non-protected way (Elshaghabee et al., 2017). Our *in vitro* studies revealed a high tolerance of BTS1-KO *S. cerevisiae* to gastric juice acidity and bile salts. Moreover, probiotics are mainly consumed in the presence of milk proteins in clinical settings owing to their protective effect on probiotics, and these milk proteins support the survival of probiotics in the acidic environment of the stomach. Even though BTS1-KO *S. cerevisiae* showed decreased viability at low pH *in vitro*, they exhibited substantial viability when consumed as adjuncts in a matrix of fermented milk (Zhang et al., 2011). These findings underscore the clinical potential of BRS1-KO *S. cerevisiae* as a probiotic.

Conventional antibiotics primarily target pathogenic bacteria by interfering with their cell wall (penicillins and cephalosporins), cell membranes (polymyxins), protein synthesis (tetracyclines and chloramphenicol), nucleic acid replication and transcription (rifampin) (Abushaheen et al., 2020). Biofilm is an important virulence factor for bacteria, greatly increasing their virulence and drug resistance (Rabin et al., 2015). Hence, inhibiting bacterial biofilm formation is a promising therapeutic strategy. This study demonstrated that BTS1-KO *S. cerevisiae* inhibited *E. coli and S. aureus* biofilm formation *in vitro* at concentrations lower than MIC, along with exerting a curative effect on the infected larvae of *G. mellonella in vivo*.

The formation of biofilm is a complex process involving bacterial attachment, colony formation, maturation and diffusion (Yang et al., 2012). Among these stages, adhesion to the carrier surface is the most critical step (Berry et al., 2022). Studies report that biofilms are composed of cellular biomass (10%) and EPS (90%), which facilitate bacterial adhesion and aggregation to the carrier surface (Carniello et al., 2018). The dense structure of EPS constitutes a physiological barrier to the penetration of antimicrobial drugs, which, coupled with the fact that antimicrobial drugs are often trapped and retained by EPS, greatly weakens the bactericidal effect on the biofilm. Therefore, targeting EPS presents an effective strategy for inhibiting biofilm formation (Jayathilake et al., 2017). For instance, the antimicrobial peptide S4 (1-16) M4Ka exerts anti-biofilm activity against *P. aeruginosa* by disintegrating membrane lipids, dispersing bacteria and inhibiting biofilm formation (Quilès et al., 2016). Additionally, bacterial hydrophobicity could help bacteria resist external harmful substances and promote bacterial adherence to the carrier surface (Cannon et al., 2017; Simon et al., 2021). In this study, we found that BTS1-KO *S. cerevisiae* greatly weakened the adhesion ability and EPS production of *E. coli* and *S. aureus.* BTS1-KO *S. cerevisiae* also significantly reduced the hydrophobicity of the cell surface of *E. coli*, but not that of *S. aureus*, which could be attributed to differences in their cell wall composition. The gram-negative cell wall is mainly composed of lipopolysaccharides, with significantly enhanced hydrophobicity. These findings suggest that BTS1-KO *S. cerevisiae* inhibits biofilm formation by reducing adhesion capacity, EPS production and bacterial hydrophobicity, thereby attenuating their virulence.

In previous studies, individual differences in probiotic yeast were evident, which resulted in differences in bacterial inhibitory abilities between strains of the same genus. The antimicrobial ability of yeast might be related to the production of extracellular proteases, toxin proteins, organic acids (lactic, acetic and butyric acid), sulphur dioxide and antimicrobials (Fakruddin et al., 2017; Afroz et al., 2021). For example, lysosomes isolated from *S. cerevisiae* treated with H_2_O_2_ can also be used as antimicrobial agents with effective antimicrobial activity against *E. coli*, *Xanthomonas oryzae*, *Shigella flexnery*, *Streptomyces albus* and *Deinococcus radiophilus* (Yoon et al., 2009). Furthermore, *S. boulardii* inhibits the growth of *Candida albicans* through the production of caprylic acid (Murzyn et al., 2010). When heat-inactivated cells were used in place of live BTS1-KO *S. cerevisiae* in *G. mellonella* infection model, the survival of treated *G. mellonella* was similar to that of the control group, indicating that metabolically active BTS1-KO *S. cerevisiae* was required for effective protection.

The enzyme encoded by *BTS1* gene plays a key role in terpenoid biosynthesis in *S. cerevisiae*, catalysing the synthesis of geranyl geranyl pyrophosphate (GGPP) from substrates, which is a precursor of many terpenes, such as carotenoids (Jiang et al., 1995). Farnesene belongs to terpenoids, and its synthetic precursor is farnesyl pyrophosphate (FPP). The synthesis of GGPP catalysed by BTS1 and FPP share some upstream metabolic intermediates and enzymatic reactions, so the activity and expression level of BTS1 will indirectly affect the content of FPP in cells, and then affect the synthesis of farnesene. A prior study reported that the deletion of BTSI can change the metabolic flux of GGPP and FPP in cells, and make more precursors flow to the synthetic pathway of farnesene, thus increasing the yield of farnesene (Wang et al., 2022). Certain terpenoids have been reported to possess antibacterial activity; however, in this study, we found that farnesene exerted no antibacterial effect. Organic acid production is a common mechanism underlying probiotic antibacterial activity, with acids acidifying the growth environment to inhibit bacterial pathogen’s proliferation (Evivie et al., 2019). For instance, the undissociated form of lactic acid permeabilises gram-negative bacteria’s outer membranes, leading to intracellular acidification and bacteriostatic activity (Asmat et al., 2018). In our study, we found that the pH of BTS1-KO *S. cerevisiae* CFS was reduced compared to the parent strain. The antimicrobial activity disappeared when the pH of BTS1-KO *S. cerevisiae* CFS was adjusted to 6.5. Furthermore, UPLC-MS/MS analysis revealed that BTS1-KO *S. cerevisiae* produced high contents of lactic acid, up to 1130.55 ug/mL. These indicated that BTS1-KO *S. cerevisiae* inhibits the growth and virulence of pathogenic bacteria by increasing the production of lactic acid or that the antimicrobial active substances produced by BTS1-KO *S. cerevisiae* could only exert antibacterial effect in an acidic environment. It is reported that lactic acid bacteria are one of the most important probiotics. They mainly produce antibacterial effects by fermenting carbohydrates and producing a large amount of lactic acid. The concentration of lactic acid in the supernatant produced by lactic acid bacteria fermentation is between 2.41 and 7.12 g/L. Although the content of lactic acid in the supernatant of BTS1-KO *S. cerevisiae* is lower than that of lactic acid bacteria, the results of the antibacterial spectrum experiment showed that the antibacterial effect of BTS1-KO *S. cerevisiae* is equivalent to that of lactic acid bacteria (Greifová et al., 2017; Qadi et al., 2023). In addition, the application of BTS1-KO *S. cerevisiae* during antibiotic treatment has certain advantages over bacterial probiotics because due to its natural characteristics as a fungus, it has inherent resistance to antibiotics and cannot promote the spread of antibiotic resistance. Studies have shown that lactic acid, a metabolic by-product of host and intestinal microbiota, is generally considered a safe and stable compound (Nilsson et al., 1999). Therefore, it can be considered that BTS1-KO *S. cerevisiae* can stably exert antibacterial effect. Further analysis of required to elucidate whether BTS1-KO *S. cerevisiae* can produce additional active metabolites to inhibit pathogens.

## 5 Conclusion

BTS1-KO *S. cerevisiae* demonstrated potent antibacterial activity against different pathogens without cytotoxic effects both *in vitro* and *in vivo*. It achieves this by inhibiting biofilm formation through reductions in adhesion and EPS production. Moreover, BTS1-KO *S. cerevisiae* exhibits therapeutic efficacy in infected *G. mellonella larvae in vivo*. Mechanistic investigations reveal that increased lactic acid production plays a significant role in its antibacterial activity. These findings highlight the potential of BTS1-KO *S. cerevisiae* as a probiotic and offer a promising approach for managing bacterial infections.

## Data Availability

The datasets presented in this study can be found in online repositories. The names of the repository/repositories and accession number(s) can be found in the article/supplementary material.
